# ADAMTS-13 in the Diagnosis and Management of Thrombotic Microangiopathies

**DOI:** 10.5041/RMMJ.10160

**Published:** 2014-10-29

**Authors:** Galit Sarig

**Affiliations:** Hematology Laboratory, Rambam Health Care Campus; and Bruce Rappaport Faculty of Medicine, Technion, Israel Institute of Technology; Haifa, Israel

**Keywords:** ADAMTS-13, aHUS, HUS, thrombotic microangiopathies, TTP, UL-VWF, Von Willebrand factor

## Abstract

Thrombotic microangiopathies (TMAs) comprise a group of distinct disorders characterized by microangiopathic hemolytic anemia, thrombocytopenia, and microvascular thrombosis. For many years distinction between these TMAs, especially between thrombotic thrombocytopenic purpura (TTP) and hemolytic uremic syndrome (HUS), remained purely clinical and hard to make. Recent discoveries shed light on different pathogenesis of TTP and HUS. Ultra-large von Willebrand factor (UL-VWF) platelet thrombi, resulting from the deficiency of cleavage protease which is now known as ADAMTS-13 (a disintegrin and metalloproteinase with a thrombospondin type 1 motif, member 13), were found to cause TTP pathology, while Shiga toxins or abnormalities in regulation of the complement system cause microangiopathy and thrombosis in HUS. TMAs may appear in various conditions such as pregnancy, inflammation, malignancy, or exposure to drugs. These conditions might cause acquired TTP, HUS, or other TMAs, or might be a trigger in individuals with genetic predisposition to ADAMTS-13 or complement factor H deficiency. Differentiation between these TMAs is highly important for urgent initiation of appropriate therapy. Measurement of ADAMTS-13 activity and anti-ADAMTS-13 antibody levels may advance this differentiation resulting in accurate diagnosis. Additionally, assessment of ADAMTS-13 levels can be a tool for monitoring treatment efficacy and relapse risk, allowing consideration of therapy addition or change. In the past few years, great improvements in ADAMTS-13 assays have been made, and tests with increased sensitivity, specificity, reproducibility, and shorter turnaround time are now available. These new assays enable ADAMTS-13 measurement in routine clinical diagnostic laboratories, which may ultimately result in improvement of TMA management.

## INTRODUCTION

Thrombotic microangiopathies (TMAs) comprise a group of distinct disorders characterized by microangiopathic hemolytic anemia, thrombocytopenia, and microvascular thrombosis, regardless of cause or specific tissue involvement.^1^ The pathogenesis is associated with thrombus formation in the micro-vasculature of various organs, which leads to a consumptive thrombocytopenia and creates an abnormally high level of shear stress in the small vessels.^2^,^3^ The shear stress eventually leads to mechanical destruction of erythrocytes and the presence of fragmented erythrocytes (schistocytes) in the peripheral blood.

Thrombotic microangiopathies may result from four types of lesions: ultra-large von Willebrand factor (UL-VWF)-platelet thrombi, as in thrombotic thrombocytopenic purpura (TTP); fibrin-platelet thrombi, as exemplified by disseminated intravascular coagulopathy (DIC) and catastrophic antiphospholipid syndrome; inflammatory or proliferative microangiopathy accompanied by variable fibrin thrombi, as in hemolytic uremic syndrome (HUS); and intravascular clusters of cancer cells.^4^

For many years, the distinction between these TMAs remained purely clinical, and this led to the belief that these disorders were different manifestations of the same pathological process. However, recent advances have demonstrated that UL-VWF-platelet thrombi in TTP result from the deficiency of a VWF cleavage protease which is now known as ADAMTS-13 (a disintegrin and metalloproteinase with a thrombospondin type 1 motif, member 13), whereas microangiopathy and thrombosis as in HUS result mainly from exposure to Shiga toxins or abnormalities in regulation of the complement system.^4^

To understand differences in pathophysiology of TMAs, this review describes the history of TMA and ADAMTS-13 discovery, various TMAs, and the way to differentiate between them. In addition, the article discusses the importance of rapid ADAMTS-13 evaluation to ensure an accurate diagnosis and urgent initiation of the appropriate therapy. The role of ADAMTS-13 status in risk assessment and monitoring response to treatment is also addressed.

## THE HISTORY OF TMA PATHOGENESIS DISCOVERY—FROM BEDSIDE TO BENCH

The history of the TMA discovery (between 1924 and 1960) is associated with very talented clinicians who had the ability and the vision to recognize the pathophysiology of the diseases, although they lacked the technology to demonstrate and prove it. Not until the 1980s was evidence for the proposed mechanisms discovered.

In 1924, Eli Moschcowitz was the first to report on a 16-year-old girl with a sudden onset of fever and hemolytic anemia, followed rapidly by paralysis, coma, and death. Moschcowitz suspected that microvascular platelet-rich thrombi which were found in the microcirculation were the cause for this disease.^5^ This was probably the first description of TTP.

In 1955, Gasser et al. described five childhood cases of HUS, which were clinically defined by thrombocytopenia, non-immune microangiopathic hemolytic anemia, and acute kidney failure followed by death from renal cortical necrosis.^6^

In 1960, Schulman et al. described the case of an 8-year-old girl who exhibited relapsing episodes of thrombocytopenia. This patient responded well to plasma infusion, and the authors suggested that the disorder was due to the deficiency of a platelet-stimulating factor. Upshaw later reported comparable findings in a 29-year-old woman whose first episode occurred at the age of 6 months. The author suggested as the underlying pathogenic mechanism the deficiency of a plasma factor that promotes platelet and red blood cell destruction.^7^ It is now clear that the disorder described by Schulman and Upshaw, which now bears their name—the Upshaw–Schulman syndrome—represents a congenital form of TTP.

In 1982, Moake et al. found ultra-large molecular forms of von Willebrand Factor (UL-VWF) in patients with TTP and proposed that this played a pathogenic role in the formation of microvascular platelet-rich thrombi in patients with acute TTP.^8^

In 1985, Karmali et al. discovered the link between the HUS and enteric infections with *Escherichia coli* that produce Shiga toxin (Stx).^9^

In 1996, simultaneously, Furlan et al. in Switzerland and Tsai in New York reported independently the isolation and identification of a VWF-cleaving protease from human plasma.^10^,^11^

In 2001, several groups (Fujikawa et al., Gerritsen et al., and Levy et al.) identified the VWF-cleaving protease as ADAMTS-13.^12^–^14^

**Figure 1.**
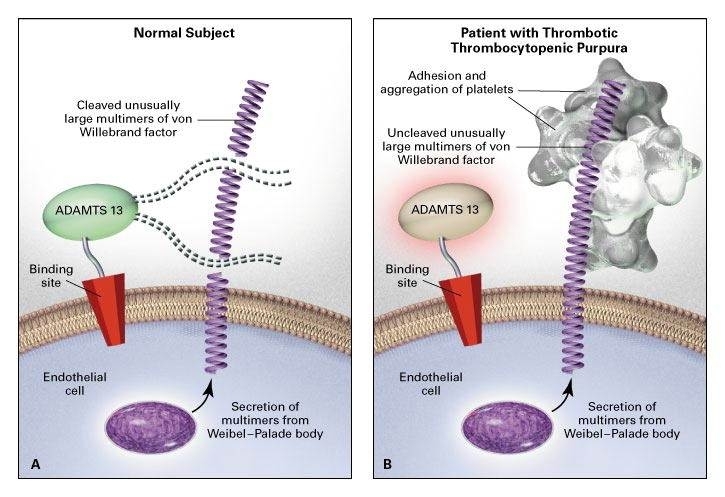
**Proposed Relation among the Absence of ADAMTS-13 Activity *In Vivo*, Excessive Adhesion and Aggregation of Platelets, and Thrombotic Thrombocytopenic Purpura.** In Panel A, in normal subjects, ADAMTS-13 (von Willebrand factor–cleaving metalloprotease) molecules attach to binding sites on endothelial cell surfaces and cleave unusually large multimers of von Willebrand factor as they are secreted by stimulated endothelial cells. The smaller von Willebrand factor forms that circulate after cleavage do not induce the adhesion and aggregation of platelets during normal blood flow. The ADAMTS-13 may use one of its thrombospondin-1–like domains or its arginine–glycine–aspartate (RGD) sequence to attach to the surface of endothelial cells. In Panel B, absent or severely reduced activity of ADAMTS-13 in patients with thrombotic thrombocytopenic purpura prevents timely cleavage of unusually large multimers of von Willebrand factor as they are secreted by endothelial cells. The uncleaved multimers induce the adhesion and aggregation of platelets in flowing blood. A congenital deficiency of ADAMTS-13 activity or an acquired defect of ADAMTS-13 (such as that caused by autoantibodies or by a change in the production or survival of the protein) can lead to thrombotic thrombocytopenic purpura. Interference with the attachment of ADAMTS-13 to endothelial cells *in vivo* (for example, as a result of ADAMTS-13–receptor blockade by other types of autoantibodies) may also cause thrombotic thrombocytopenic purpura in patients with normal ADAMTS-13 activity in plasma. From: Moake JL. Thrombotic microangiopathies. N Engl J Med 2002;347:587–600. Copyright © Massachusetts Medical Society. Reprinted with permission from Massachusetts Medical Society.

The majority of patients with TTP show severe deficiency in the VWF-cleaving activity of ADAMTS-13, either caused by missense or frame-shift mutations^14^–^16^ (Figure 1B) or due to ADAMTS-13 neutralizing autoantibodies.^17^,^18^

An ADAMTS-13 deficiency and defective complement regulation have been identified as the two major causes of TMA. It is now possible to classify TMA pathogenetically rather than just clinically.

Recently, it was shown that the coagulation cascade, platelet activation, ADAMTS-13 activity, and UL-VWF multimers are associated with the complement pathway regulation.^19^ The role of these interrelationships in TMAs should be further studied to improve TMA management.

## VON WILLEBRAND FACTOR AND ULTRA-LARGE MULTIMERS OF VWF

Von Willebrand factor (VWF) is a large complex of multimeric molecules, range 0.8–20 × 10^6^ kDa, encoded on chromosome 12. Synthesis of VWF is by endothelial cells and megakaryocytes and stored in Weibel–Palade bodies in endothelial cells and in platelet α granules. Secretion is induced by vascular injury and stimuli by thrombin, histamine, vasopressin, inflammatory cytokines, and Stx.

Von Willebrand factor is released from endothelial cells as ultra-large multimers of VWF (UL-VWF) which can bind the glycoprotein (GP) Ibα components of platelet GPIb-IX-V receptor and induce platelet adhesion and aggregation by shear stress in the arterioles and capillaries (Figure 1B).^20^,^21^ These microvascular thrombi of platelets with UL-VWF result in platelet consumption and hemolysis and cause thrombotic microangiopathy of several TMAs.^22^

In normal subjects, the UL-VWF molecules are cleaved by a specific VWF-cleaving metalloprotease present in the plasma, ADAMTS-13 (Figure 1A).^10^–^14^ The enzyme degrades UL-VWF multimers by cleaving 1605Tyr–1606Met peptide bonds in susceptible A2 domains of VWF monomeric subunits.^1^

## THE STRUCTURE OF ADAMTS-13

**Figure 2.**
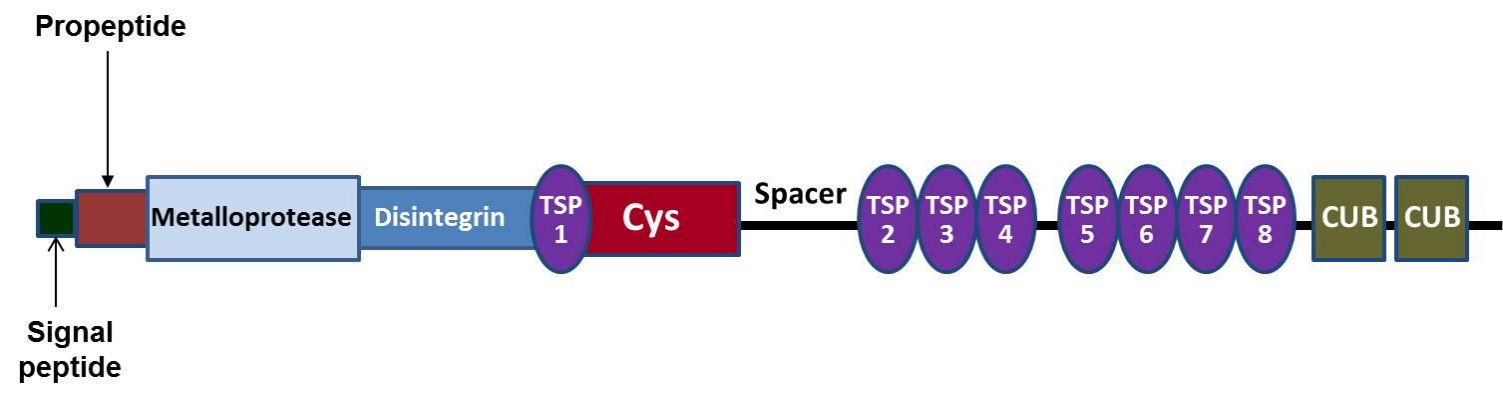
**ADAMTS-13 Domain Structure.** ADAMTS-13 N-terminal composed of a signal peptide and propeptide, which are cleaved during the protein processing. The catalytic domain consists of the metalloprotease and disintegrin domains. A first thrombospondin-1 (TSP 1) is followed by a cysteine-rich domain (Cys), a spacer, and sevenadditional TSP 1 repeats (TSP 2–8). The C terminus contains two complement components C1r/C1s, embryonic sea urchin protein, and bone morphogenic protein-1(CUB) domains.

ADAMTS-13 is a disintegrin and metalloprotease with eight thrombospondin-1-like domains (Figure 2) composed of an amino-terminal reprolysin-type metalloprotease domain followed by a disintegrin domain, a thrombospondin-1-like domain, a cysteine-rich domain containing an arginineglycine-aspartate sequence and an adjacent spacer portion, seven additional thrombospondin-1-like domains, and two similar CUB domains at the carboxyl-terminal end of the molecule. The CUB domains, found only in ADAMTS-13 among the ADAMTS enzyme family members, contain peptide sequences present in complement subcomponents C1r/C1s, embryonic sea urchin protein, and bone morphogenic protein-1.^23^

The spacer and CUB-1 domains are involved in binding ADAMTS-13 to the UL-VWF secreted by endothelial cells.^24^ The ADAMTS-13 enzymes attach through spacer/CUB domains to accessible A3 domains in the monomeric subunits of the VWF strings,^25^ and then cleave a 1605Tyr–1606Met peptide bond in an adjacent VWF A2 domain.

ADAMTS-13 is a Zn^2+^- and Ca^2+^-requiring 190,000-dalton glycosylated protein that is encoded on chromosome 9q34 and produced predominantly in the liver. The activity of ADAMTS-13 is inhibited *in vitro* by ethylenediaminetetra-acetic acid (EDTA), and, therefore, functional assays of the enzyme are usually performed using plasma anticoagulated with citrate.^10^,^11^ Anti-ADAMTS-13 antibodies preferentially bind to the cysteine-rich and spacer regions of the ADAMTS-13 molecule.

## THROMBOTIC MICROANGIOPATHY

Thrombotic microangiopathy (TMA) refers to a group of pathological disorders that are characterized by hemolytic anemia, thrombocytopenia, and widespread microvasculopathy, with or without thrombi.^4^

Clinical manifestations of TMA reflect ischemic injury of the affected organs. In some patients neurological deficits predominate; in others, renal failure is severe. This clustering provided a convenient basis for defining thrombotic thrombocytopenic purpura (TTP) and the hemolytic uremic syndrome (HUS). However, this classification has been misleading, since some patients have both neurological deficits and renal failure, and others may have predominant neurological deficits or renal failure on different occasions.

Thrombotic microangiopathy may appear in a variety of conditions such as pregnancy, inflammation, malignancy, or exposure to such drugs as thienopyridines or calcineurin inhibitors. These conditions might be the cause of acquired TTP, HUS, or HELLP syndrome (hemolysis, elevated liver enzymes, low platelets), or the trigger in individuals with a genetic predisposition to ADAMTS-13 or complement factor H deficiency.

While patients with congenital TTP and acquired immune TTP attributed to low ADAMTS-13 activity demonstrate a good response to plasma infusion or plasma exchange (PEX), other clinical forms of TMA occur in the absence of severe ADAMTS-13 deficiency, and this may be the reason why patients with the other clinical forms of TTP do not respond to plasma therapy.^26^

The diagnosis of TMA can be very difficult, as there is a clinical overlap between various TMAs. Since in untreated cases mortality may approach 90%, the availability of ADAMTS-13 activity and anti-ADAMTS-13 antibody assays is crucial for the differentiation between the TMAs, accurate diagnosis, and urgent initiation of the appropriate treatment.

### Thrombotic Thrombocytopenic Purpura

Thrombotic thrombocytopenic purpura (TTP) is rare, with a reported incidence of 4–6 cases per million per year and with a female-to-male ratio of 3:2.^27^,^28^ In spite of major progress in early detection and modern therapies, early death still occurs: approximately half of the deaths in the regional UK TTP registry occurred within 24 hours of presentation.^27^

Thrombotic thrombocytopenic purpura is characterized by microvascular platelet clumping, resulting in microangiopathic hemolytic anemia, fragmented erythrocytes (schistocytes), consumptive thrombocytopenia, renal dysfunction, and neurological symptoms. However, TTP can present without the full pentad; up to 35% of patients do not have neurological signs at presentation, and renal abnormalities and fever are not prominent features. The revised diagnostic criteria state that TTP must be considered in the presence of thrombocytopenia and microangiopathic hemolytic anemia alone.^29^ The diagnosis of TTP remains based on clinical history, examination of the patient, and the blood film. Assays for ADAMTS-13 help to confirm the diagnosis, differentiate TTP from other TMA forms, and monitor the efficacy of treatment. They are also helpful in consideration of the need for additional or alternative therapy.

In general, congenital TTP is defined by ADAMTS-13 deficiency, while acquired TTP is defined by the presence of ADAMTS-13 neutralizing autoantibodies. During an acute episode, before starting therapy, ADAMTS-13 activity level of <5% supports the TTP diagnosis.

### Congenital TTP: Upshaw–Schulman Syndrome

This very rare condition with a prevalence of about 0.05–0.4 per 100,000 is transmitted by autosomal recessive inheritance.^30^,^31^ Neonates with severe phenotype typically have major neonatal jaundice. Blood film examination may show schistocytes together with red cell anisocytosis.^32^ More frequently, the diagnosis is made later in infancy or childhood,^33^ typically with thrombocytopenia, microangiopathic hemolytic anemia, jaundice, and elevated lactate dehydrogenase (LDH), although some children may only have an isolated thrombocytopenia. Neurological symptoms, such as hemiparesis, hemiplegia, or seizures, occur in 35% of cases.^34^

Patients presenting during adulthood tend to have milder clinical courses.^31^ However, the clinical course in individual patients can be highly variable.^30^ The presence of an affected sibling or a therapeutic response to a plasma-containing blood product may propose the diagnosis.^35^

The diagnosis of congenital TTP is dependent on detecting ADAMTS-13 activity of <5% during an acute episode, in the absence of antibodies to ADAMTS-13. Over the last few years, molecular analysis has been used to confirm the diagnosis, and either a homozygous or compound heterozygote defect in ADAMTS-13 could be found. Testing of siblings and other first-degree relatives at risk should be considered.^36^

Mutations affecting the highly conserved N-terminal domains of ADAMTS-13 are associated with lower residual ADAMTS-13 activity and a more severe clinical phenotype in an allelic dose-dependent manner.^37^,^31^ Mutations located in the C-terminal part of ADAMTS-13 are associated with a less severe clinical expression.

A mutation located in the TSP1–7 domain, p.Arg1060Trp, is specifically associated with an adult onset of congenital TTP and found with a very high prevalence in women in whom TTP events are associated with pregnancy.^38^

Patients with congenital TTP have persistently low levels of ADAMTS-13, but they can be asymptomatic until a further precipitating event results in an acute TTP episode. Events include febrile episodes, infections, vaccinations, excess alcohol intake, and in females mostly pregnancy.^39^–^41^

Rarely, patients with a “late-onset phenotype” may not develop symptoms until their 50s or 60s with isolated cerebral events or renal disease.^42^ Asymptomatic male cases are usually detected because they have affected siblings.

### Acquired Thrombotic Thrombocytopenic Purpura

Acquired TTP is a rare, autoimmune disease characterized by antibodies, usually IgG, directed against ADAMTS-13, with an annual incidence of 0.2–1 per 100,000.^43^ In its most common, characteristic form, TTP begins abruptly and virulently, occasionally after a febrile, viral-like prodrome; a minor infection or pregnancy may be the trigger.^44^,^45^ Thrombocytopenia and fragmentation hemolysis are severe, and central neurologic signs exist at presentation or supervene quickly, out of proportion to renal signs. Dialysis-requiring renal failure is rare. Without immediate recognition and intervention, death, often precipitated by seizures and arrhythmias, may come rapidly and suddenly. Before the advent of modern therapy, mortality was about 90%. One-third of TTP survivors experience relapses over the course of years, especially soon after initial presentation.^46^,^47^ Some have persistent cognitive and central neurologic impairments, other chronic health problems, or die prematurely even when TTP is inactive.^48^–^50^ About 5%–10% of patients later in their course manifest systemic lupus erythematosus.^50^

### Hemolytic Uremic Syndrome

Hemolytic uremic syndrome (HUS) is a TMA defined by thrombocytopenia, microangiopathic hemolytic anemia, and acute renal failure with elevated serum creatinine levels, low glomerular filtration rates, microscopic hematuria, and subnephrotic proteinuria.^51^ The most frequent form is associated with infections by Shiga-like toxin-producing bacteria (Shiga-HUS). Atypical form of HUS (aHUS) is associated with defects in the immunological complement pathway.

Shiga toxins (Stx)-1 and Stx-2, produced by enterohemorrhagic *E. coli*, stimulate rapid and profuse secretion of UL-VWF from endothelial cells, in particular glomerular microvascular endothelial cells.^53^ Platelets immediately adhere to the secreted UL-VWF, and the rate of platelet–VWF string cleavage by ADAMTS-13 is delayed in the presence of Stx-1 or Stx-2. This may explain the glomerular microvascular occlusion and acute renal failure.^52^

Hemolytic uremic syndrome usually occurs as a single episode, except for rare individuals who have a familial, recurrent type of the disease.^1^,^23^ In these patients, often children, with aHUS, the level of the plasma complement control protein, factor H, is abnormally low most of the time. The result is over-activation of complement component 3 (C3), which causes permanent glomerular endothelial activation and obstruction of the glomerular microvasculature by platelet–fibrin thrombi.^23^

### Shiga Toxin-induced HUS

About 10%–20% of symptomatic infections lead to HUS.^23^,^53^ Shiga toxin-induced HUS (Shiga-HUS) is the commonest TMA, most prevalent in children under the age of 5 years, with an annual incidence of 6 per 100,000.^54^ Severe thrombocytopenia, fragmentation hemolysis, renal failure, and hyper-tension are characteristic.

The diagnosis of Shiga-HUS depends on the detection of *E. coli* O157:H7 and other Stx-producing bacteria and their products in stool cultures.^51^

In extreme cases, the brain and other organs may be involved. The condition is a medical emergency with a short-term mortality of about 5%–10% without urgent therapy. Renal function recovers in 70% to over 90% of cases.^35^

Approximately 5% of HUS cases in children are not associated with Stx-producing bacteria and result from infection by neuraminidase-producing *Streptococcus pneumoniae*.^51^

### Atypical HUS

Cases of atypical HUS (aHUS) are rare, one-tenth as frequent as Shiga-HUS.^54^ The first presentations are most of the time in children, including neonates, but may not occur until later in life. About 20% are familial phenomena. Infections and pregnancy may trigger an acute episode.^54^ The end-organ presentation is predominantly renal, but cardiac, neurologic, and more rarely large artery obstruction may occur. The prognosis before recent treatment advances was poorer than for Shiga-HUS, with over half of cases progressing to end-stage renal failure and one-quarter of patients dying of the disease.^35^

Atypical HUS is the result of excessive alternative complement pathway activation.

Prominent causes of aHUS are a heterozygous mutation of the complement factor H gene, or homozygous deletion in genes for the factor H-related proteins or autoantibody-mediated inhibition of factor H deficiency.

Other mutations associated with aHUS include heterozygous loss-of-function mutations of complement factor I, membrane co-factor protein or CD46 or thrombomodulin, heterozygous gain-of-function mutations in C3 or complement factor B.

Excessive alternative complement pathway activity in aHUS results in severe damage of renal endothelial cells, leading to renal failure.^19^

Lately, treatment with eculizumab has been approved for aHUS. Eculizumab is a humanized monoclonal antibody to terminal complement protein C5 that prevents activation of the terminal complement pathway by binding C5 and inhibiting generation of pro-inflammatory C5a and the lytic C5b-9 membrane attack complex. Before administering eculizumab therapy for an acute episode of aHUS, there is a need to rule out TTP which is proved by normal levels of ADAMTS-13 activity (>30%), without the presence of anti-ADAMTS-13 antibodies.

### Pregnancy-associated TMA

Pre-eclampsia and HELLP syndrome are serious TMA complications in pregnancy. In these events, the hypoxic placenta releases receptors for angiogenic factors, like soluble VEGF receptor-1. These circulating soluble angiogenic receptors contribute to the progressive renal dysfunction and hepatic necrosis in pregnancy TMAs.^55^

Nevertheless, pregnancy is a hypercoagulation state with very high levels of VWF and UL-VWF released from endothelial cells and the placenta, and can trigger TTP (acquired or congenital), aHUS, or other TMAs.

Pregnancy is the initiating event for approximately 5%–25% of TTP cases, which are late-onset adult congenital TTP or acute idiopathic TTP.^27^,^36^

Thrombosis occurs in the placenta in untreated TTP pregnancies, resulting in fetal growth restriction, intrauterine fetal death, and pre-eclampsia. There is a continued risk of relapse during subsequent pregnancies. However, there are some reports demonstrating that women with normal pre-pregnancy levels of ADAMTS-13 have a lower risk of relapse.^56^,^57^ Differentiating TTP from more common pregnancy-related TMAs is difficult. In these suspected cases, measurement of ADAMTS-13 activity and anti-ADAMTS-13 antibodies can advance the differentiation. Low ADAMTS-13 activity and the presence of anti-ADAMTS-13 antibodies can distinguish congenital and acquired TTP, respectively, from other pregnancy-associated TMAs. Although in pre-eclampsia and HELLP syndrome ADAMTS-13 activity is reduced (median 31%, range 12%–43%), in an acute TTP episode the ADAMTS-13 activity levels are below 10%. In addition, presence of anti-ADAMTS-13 antibodies supports the diagnosis of acquired TTP.^36^

### TMA Due to Drugs

Thienopyridines (ticlopidine and clopidogrel) are the most frequent TMA-causing drugs reported to the United States Food and Drug Administration. Ticlopidine, now infrequently prescribed, produced the highest incidence, 1 in 1,600 to 5,000 patients. About 90% of cases occurred between 2 and 12 weeks of therapy. Clopidogrel causes TMA less frequently, about 1 in 80,000 patients.^58^,^59^ Other drug causes are the calcineurin inhibitors (cyclosporine and tacrolimus), the mTOR inhibitors (sirolimus and everolimus), anti-neoplastic agents (mitomycin and gemcitabine, both in a cumulative, dose-dependent manner), and quinine.^35^

Drugs appear to be responsible for <15% of all TTP cases. Ticlopidine therapy increases the risk of developing ADAMTS-13 inhibitors 200- to 300-fold.^60^,^61^

Some chemotherapy agents, such as gemcitabine, bleomycin, and mitomycin-C, can cause HUS but not TTP.^36^

### Transplant and Malignancy-associated TMA

Transplant-associated TMA is a microangiopathy hemolytic anemia with thrombocytopenia that occurs after bone marrow transplantation. It may reflect endothelial toxicity associated with chemotherapy, infections, immunosuppressives, and graft-versus-host disease (GVHD).^35^,^62^

Thrombotic microangiopathy occurs in association with a variety of malignancies, especially adeno-carcinomas.^63^ Presentation may be either at an early stage of cancer or associated with disseminated disease.

The ADAMTS-13 activity is not significantly reduced in transplant and malignancy-associated TMA,^64^ and this might explain the inefficiency of PEX therapy in these patients. Some suggest that due to endothelial damage associated with malignancy, chemotherapy, and bone marrow transplantation, there is an extreme release of UL-VWF which even normal ADAMTS-13 level cannot cleave, and this may lead to TMA events. Future drugs targeting the VWF-platelet interaction could be efficient in transplant and malignancy-associated TMA.

## ASSAYS FOR ADAMTS-13 MEASUREMENT

Several different types of assays are available for the measurement of ADAMTS-13: activity, antigen, inhibitor, and anti-ADAMTS-13 antibodies. The activity assays are most commonly reported in the literature; however, the measurement of anti-ADAMTS-13 antibodies is also highly important for the accurate TMA differentiation and TTP diagnosis (Table 1).

**Table 1.** ADAMTS-13 Levels and Pathophysiology in the Different Thrombotic Microangiopathies (TMAs).

**Type of TMA**	**Pathophysiology**	**ADAMTS-13 Level During Acute Episode**	

		**Activity**	**Anti-ADAMTS-13 Antibodies**
Congenital TTP	ADAMTS-13 deficiency causes UL-VWF-platelet thrombi which lead to multi-organ ischemic failure	< 5%	Absent
Acquired TTP	Antibodies against ADAMTS-13 cause UL-VWF-platelet thrombi which lead to multi-organ ischemic failure	< 10%	Very High
Shiga-HUS	Shiga toxin causes secretion of UL-VWF and the formation of VWF-platelet thrombi in the glomerular microvasculature which lead to acute renal failure	> 20%	Absent
aHUS	Dysregulation of the complement system, mostly due to complement factor H deficiency, leads to VWF-platelet thrombi in the glomerular microvasculature and to acute renal failure	> 20%	Absent
HELLP syndrome and pre-eclampsia	Abnormal and hypoxic placenta activates the complement and coagulation cascades which lead to thrombotic microangiopathy	> 20%	Absent
Transplant and malignancy-associated TMAs	Endothelial toxicity causes thrombotic microangiopathy	> 20%	Absent
DIC	Disseminated intravascular coagulation activation leads to thrombotic microangiopathy and multi-organ ischemic failure	> 20%	Absent
Catastrophic antiphospholipid syndrome	Antiphospholipid antibodies cause endothelial damage, thrombotic microangiopathy, and multi-organ ischemic failure	> 20%	Absent

ADAMTS-13, a disintegrin and metalloproteinase with a thrombospondin type 1 motif, member 13; aHUS, atypical hemolytic uremic syndrome; DIC, disseminated intravascular coagulation; HELLP syndrome, hemolysis, elevated liver enzymes, and low platelets syndrome; Shiga-HUS, Shiga toxin-induced hemolytic uremic syndrome; TTP, thrombotic thrombocytopenic purpura; UL-VWF, ultra-large von Willebrand factor.

Functional assays measuring ADAMTS-13 activity are based on the ability of the patient plasma to degrade VWF multimers or synthetic VWF peptides. Inhibitory autoantibodies can be titrated *in vitro* using classical mixing studies, and neutralizing or non-neutralizing antibodies can be detected by Western blotting or enzyme-linked immunosorbent (ELISA) assays.^65^

Over the past few years, significant improvements have been made in ADAMTS-13 assays, and current commercially available tests allow more reproducible analyses with shorter turnaround time (1–4 hours), which can be conducted even in routine clinical diagnostic laboratories.^66^

### Activity Assays

The original, or first-generation, assays for ADAMTS-13 activity measured the activity directly on the cleavage products, using VWF multimer analysis, or indirectly, using either collagen-binding or ristocetin aggregation assays.^66^ These methods were very time-consuming and available only in specialized and research laboratories.

The first assay method was developed by Furlan et al.^10^ The substrate for this method was protease-free plasma multimeric VWF. Detected plasma samples were first diluted, then activated by barium chloride, mixed with substrate, and dialyzed. Reaction products were separated by sodium dodecyl sulfate (SDS) agarose gel electrophoresis followed by immunoblotting. The resolution of the ladders of the degraded VWF multimers was high and reproducible, but the method required several days to complete.^10^,^22^

Newer techniques using both direct and indirect assays have been introduced that are more suitable for a routine testing laboratory. These assays utilize peptide substrates based on the ADAMTS-13 cleavage site in VWF, which contains the peptide bond between 1605Tyr and 1606Met in the A2 domain of VWF. These substrates are either a recombinant VWFA2 or synthesized VWF73 peptides.^66^

Patient plasma is incubated with the peptide substrate, and the residual VWF or cleavage product is measured by electrophoresis, fluorescence resonance energy transfer (FRET) technique, or immunoassay. Results are available within a few hours, and the improved methods allow higher throughput and have improved precision and sensitivity.

Multicenter studies found that, while the original assays based on multimeric VWF are sensitive (3%–6% of ADAMTS-13 activity) and reproducible, the newer assays based on VWF peptides are more sensitive (1%–3% of ADAMTS-13 activity), reproducible, easier, and rapid (1–4 h).^66^,^67^

According to the 2014-2 survey of the External Quality Control for Assays and Tests with a focus on Thrombosis and Haemostasis (ECAT Foundation), the majority of participants (43/70) (61%) use ELISA method in ADAMTS-13 activity measurement with 13% coefficient of variation (CV); most of them (36/43) use the Technoclone Technozyme ADAMTS-13 activity kit. Only 24 (34%) laboratories use the FRET method with 34% CV.^68^

### Anti-ADAMTS-13 Autoantibodies

Two types of anti-ADAMTS-13 antibodies have been described: one inhibiting (neutralizing) ADAMTS-13 proteolytic activity^17^,^18^ and the other binding to the protease and accelerating its clearance from plasma through opsonization and/or other yet unresolved mechanisms.^69^ Both of these antibodies may be simultaneously present in many TTP patients.^65^

Neutralizing ADAMTS-13 autoantibodies (inhibitor) can be titrated *in vitro* using classic mixing studies of heat-inactivated patient and normal plasmas at a 1:1 dilution or several dilutions. However, although useful, Bethesda assays are far from being optimized and generally lack sensitivity. Less frequently (about 30%), autoantibodies are non-neutralizing and probably promote the clearance of ADAMTS-13 from blood without inhibiting its activity.^69^ These non-neutralizing antibodies can be detected using Western blotting or ELISA assays.^65^

New assays use recombinant ADAMTS-13 for the measurement of anti-ADAMTS-13 antibodies in a simplified ELISA. The detection wells are coated with recombinant ADAMTS-13. Antibodies against ADAMTS-13 from the detected plasma sample are recognized by conjugated anti-human IgG. The peroxidase level is determined by a chromogenic reaction that is proportional to the anti-ADAMTS-13 antibody level. The time for results with this method using the Technozyme ADAMTS-13 INH kit, (Technoclone, Vienna, Austria) is 2 hours 15 minutes.

Of 26 laboratories that participated in the 2014-2 survey of the ECAT Foundation, 19 (73%) perform the anti-ADAMTS-13 antibodies measurement using the Technoclone Technozyme ADAMTS-13 INH kit,^68^ which is suitable for a rapid diagnosis in the routine clinical laboratory.

### Pre-analytical Variables of ADAMTS-13 Measurement Assays

Blood samples for ADAMTS-13 activity and antibody should be drawn prior to treatment initiation, for the accurate assessment of baseline ADAMTS-13 levels. Knowledge of the timing of the treatment regime in terms of sample collection is important for the appropriate interpretation of ADAMTS-13 results. Plasma infusion of even one unit, which is often done in an attempt to stabilize patients with suspected TTP, may significantly change ADAMTS-13 levels.^70^

Samples should be collected into buffered sodium citrate anticoagulant tubes and should be centrifuged within 2 hours after collection for best results. Platelet-poor plasma should be tested after centrifuging at 3,000*g* for 10 minutes or 2,000*g* for 15 minutes. If plasma is not tested within 4 hours of collection, it should be re-centrifuged and separated off into a secondary aliquot tube for storage at below −30°C for up to 3 months or below −70°C for a longer period of time. Frozen plasma samples should be thawed rapidly at 37°C for 10 minutes in a water bath, mixed thoroughly before testing, and assayed within 4 hours. If not tested immediately after thawing, samples should be kept stored at 2–8°C.^66^ Thawed samples should not be refrozen.

### The Need for ADAMTS-13 Measurement

To make the accurate diagnosis of TTP and to differentiate it from other TMAs, blood samples must be drawn during the acute episode, prior to treatment initiation, since decreased levels of ADAMTS-13 activity (20% < ADAMTS-13 < 50%) can be detected also in other TMA events (Table 1). Severely reduced ADAMTS-13 activity (<5%) during an acute episode, without presence of anti-ADAMTS-13 antibodies, supports the diagnosis of congenital TTP; whereas low ADAMTS-13 activity, in the presence of anti-ADAMTS-13 antibodies, confirms the diagnosis of acquired TTP.^36^

The specificity of severe ADAMTS-13 deficiency (<5%) in distinguishing acute TTP from HUS is 90%.^36^,^71^ This differentiation should be performed rapidly for the appropriate initiation of PEX for TTP and eculizumab for aHUS.^72^

The diagnosis of acquired TTP with severely reduced ADAMTS-13 activity and elevated level of anti-ADAMTS-13 antibodies suggests a more intensive requirement for plasma therapy, increased mortality, and the risk of refractory disease which might need further immunosuppressive therapy.^73^

### Treatment Monitoring with ADAMTS-13

While in congenital TTP it is probably sufficient to monitor plasma infusion efficiency by platelet and hemoglobin measurement only, in acquired TTP there is a benefit in monitoring the PEX efficacy by measuring the ADAMTS-13 activity and anti-ADAMTS-13 antibody levels.^74^–^76^

Persistent presence of ADAMTS-13 activity <5% with elevated anti-ADAMTS-13 antibodies during PEX therapy indicates the need for more frequent and prolonged PEX, and it may suggest the need to add immunosuppressive therapy.^77^

Plasma exchange is effective in acute TTP through replacing deficient ADAMTS-13, removing associated antibody, and reducing circulating ULVWF multimers; however, in many patients, prolonged PEX is required to achieve remission, and between 30% and 60% of patients relapse over a variable period of months to years.^73^ Therefore, the information about the ADAMTS-13 activity and antibodies can guide the decision regarding the appropriate therapy regimen for each patient.^77^

### ADAMTS-13 as Predictor for Relapse and Survival

Despite advances in TTP treatment, relapse occurs and mortality remains at 15%–20%. Patients with persistent levels of ADAMTS-13 activity of <5% and the presence of elevated levels of anti-ADAMTS-13 antibody during an acute episode, and/or during remission, have a 3-fold increased risk of relapse.^46^,^78^–^80^ Therefore, the identification of patients at the greatest risk for relapse can guide therapeutic decisions, such as the administration of prophylactic therapy or changing therapy to immunosuppressive agents to prevent relapse.^77^

To date, monoclonal anti-CD20 therapy (rituximab) appears to be the most promising immunosuppressive treatment for patients with undetectable ADAMTS-13 activity or very high anti-ADAMTS-13 antibody levels with high risk for relapse.^77^

It is noteworthy that while lower ADAMTS-13 activity in clinical remission predicts a risk of relapse, not all patients with severe ADAMTS-13 deficiency relapse. These data are consistent with previous reports suggesting that severely deficient ADAMTS-13 activity alone is not sufficient for the development of an acute episode of TTP, but rather an additional triggering factor or factors may be required. The search and identification of these triggering factors need to be continued to advance the TMAs management further.

## FUTURE PERSPECTIVES

The accurate and urgent diagnosis of TMAs is exceedingly important because of their associated organ damage and mortality.

While progress has been made in the diagnosis and treatment of TTP, further improvements in therapeutic approaches and ADAMTS-13 assay standardization are required. Several potential therapies are in development for congenital and acquired TTP: 1) Recombinant ADAMTS-13.^81^,^82^ 2) Gain-of-function ADAMTS-13 variants that resist inhibition by anti-ADAMTS-13 antibodies.^83^ 3) Drugs targeting the interaction of A1 domain of VWF with platelet GPIb such as aptamers, anti-VWF nanobodies, or monoclonal antibody.^84^–^86^ 4) Degradation or inhibition of UL-VWF.^87^ These future promising therapies are in various stages of clinical trials.

In addition, international standards for the ADAMTS-13 assays are warranted to improve test sensitivity, specificity, and reliability, as well as inter-laboratory standardization and harmonization.

## Abbreviations:

ADAMTS-13: a disintegrin and metalloproteinase with a thrombospondin type 1 motif, member 13;
aHUS: atypical hemolytic uremic syndrome;
CV: coefficient of variation;
DIC: disseminated intravascular coagulopathy;
ECAT: External Quality Control for Assays and Tests with a focus on Thrombosis and Haemostasis;
ELISA: enzyme-linked immunosorbent assay;
FRET: fluorescence resonance energy transfer;
GP: glycoprotein;
HELLP syndrome: hemolysis, elevated liver enzymes, and low platelets syndrome;
HUS: hemolytic uremic syndrome;
LDH: lactate dehydrogenase;
PEX: plasma exchange;
Stx: Shiga toxin;
TMAs: thrombotic microangiopathies;
UL-VWF: ultra-large von Willebrand factor;
VWF: von Willebrand factor.
